# Application of quality improvement strategies in 389 European hospitals: results of the MARQuIS project

**DOI:** 10.1136/qshc.2008.029363

**Published:** 2009-01-26

**Authors:** M J M H Lombarts, I Rupp, P Vallejo, R Suñol, N S Klazinga

**Affiliations:** 1 Academic Medical Center, Department of Social Medicine, University of Amsterdam, Amsterdam, the Netherlands; 2 Avedis Donabedian Institute, Autonomous University of Barcelona, and CIBER Epidemiology and Public Health (CIBERESP), Barcelona, Spain

## Abstract

**Context:**

This study was part of the Methods of Assessing Response to Quality Improvement Strategies (MARQuIS) research project investigating the impact of quality improvement strategies on hospital care in various countries of the European Union (EU), in relation to specific needs of cross-border patients.

**Aim:**

This paper describes how EU hospitals have applied seven quality improvement strategies previously defined by the MARQuIS study: organisational quality management programmes; systems for obtaining patients’ views; patient safety systems; audit and internal assessment of clinical standards; clinical and practice guidelines; performance indicators; and external assessment.

**Methods:**

A web-based questionnaire was used to survey acute care hospitals in eight EU countries. The reported findings were later validated via on-site survey and site visits in a sample of the participating hospitals. Data collection took place from April to August 2006.

**Results:**

389 hospitals participated in the survey; response rates varied per country. All seven quality improvement strategies were widely used in European countries. Activities related to external assessment were the most broadly applied across Europe, and activities related to patient involvement were the least widely implemented. No one country implemented all quality strategies at all hospitals. There were no differences between participating hospitals in western and eastern European countries regarding the application of quality improvement strategies.

**Conclusions:**

Implementation varied per country and per quality improvement strategy, leaving considerable scope for progress in quality improvements. The results may contribute to benchmarking activities in European countries, and point to further areas of research to explore the relationship between the application of quality improvement strategies and actual hospital performance.

Quality and safety of patient care are high on the European policy agenda, as evidenced by various commitments by European health ministries. Patient mobility has clearly been a triggering factor. Governments may fear that differences in the perceived quality or costs of health services may encourage patients to cross borders to obtain healthcare.^1^^–^3 The fact that member states are now talking about what is still their responsibility has increased the need for information about the cross-border movement. The Methods of Assessing Response to Quality Improvement Strategies (MARQuIS) research project aims to be instrumental in providing a better understanding of this movement, by investigating and comparing different quality improvement (QI) strategies in healthcare systems across the European Union.

In this article we focus on the degree to which QI strategies are applied at European hospitals, by their own report. As presented in the Health Care Quality Strategies in Europe study, we identified seven national QI strategies.^4^ ^5^ Our primary focus is on implementation of the strategies at the EU level; data at the country level are reported for reference purposes. The QI strategies we investigated were:

- organisational quality management programmes;
- systems for obtaining patients’ views;
- patient safety systems;
- audit and internal assessment of clinical standards;
- clinical and practice guidelines;
- performance indicators and measurements;
- external assessment.

The countries participating in this study were Spain, France, Poland, Czech Republic, the UK, Ireland, Belgium and the Netherlands.

## MATERIAL AND METHODS

### Questionnaire design

We conducted a web-based questionnaire survey. The questionnaire was developed to measure QI, defined as the application of quality policies and procedures, quality governance structures, and quality activities used to close the gap between current and expected levels of quality.^4^ To determine the distinctive aspects of QI we used several sources, such as existing QI questionnaires,^6^^–^9 a review of the quality literature,^10^^–^12 an analysis of accreditation manuals,^11^ ^13^ ^14^ and the results of previous MARQuIS studies including a literature review covering QI strategies in member states of the EU, and an analytical framework defining areas of QI policies and strategies. A glossary of quality concepts and tools was made available to participants.

The questionnaire consisted of four sections: one section focused on QI at the hospital level, the other three on quality management for specific medical conditions. The three medical conditions for focused data collection were selected based on two criteria: the condition had to represent a significant volume of cross-border patient care,^15^ and the combination of conditions was intended to cover the most relevant services offered by a hospital—that is, emergency surgical and medical services, and maternal and neonatal services. The three conditions selected were acute myocardial infarctions (AMI), acute appendicitis and deliveries. For each condition selected, the literature was searched for specific QI strategies. Search terms used included “quality assurance”, “quality improvement”, “quality assessment”, and “performance measurement”.^16^^–^31 We stopped searching when additional publications no longer resulted in new relevant QI strategies, activities or measures. Practising medical specialists were consulted for their comments and suggestions on the specific QI strategies, activities, and measures (see Acknowledgements).

**Figure 1:**
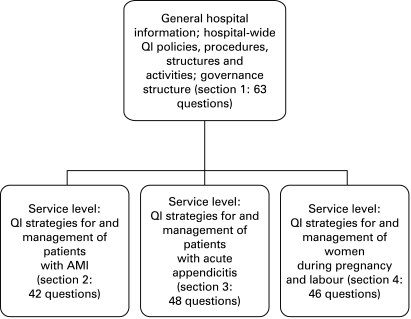
Structure of the MARQuIS questionnaire. AMI, acute myocardial infarction; QI, quality improvement.

Both members of the MARQuIS team and the nine country coordinators reviewed the draft questionnaire. (For Belgium two country coordinators were appointed, one for Flemish-speaking and one for French-speaking hospitals.) The questionnaire was then pilot tested in two hospitals in Ireland and the UK (these countries were chosen for language-related reasons), and a few amendments were made as a result. The questionnaire was translated into five languages (Spanish, French, Polish, Czech, and Dutch); the country coordinators were responsible for translation. A forward–backward translation protocol was used. Figure 1 shows the final structure of the questionnaire, which totalled 199 questions. For each of the four sections a preferred respondent (at the senior level) was suggested.

### Response categories

Various scoring scales were used depending on the type of question. Items were scored on a two-point scale (yes/no), a four-point scale (see tables 2 to 8 in Results), or a five-point Likert scale (1 =  strongly agree, 5 =  strongly disagree).

**Table 2:** Quality improvement (QI) strategies as applied in European hospitals: organisational quality management programmes/activities; total and per country*, numbers are positive responses in valid percentages (total item response in absolute numbers)

Specification of QI strategy	Total	Ireland	Belgium	France	Spain	Poland	Czech Republic
The use of ISO in implementing a quality system	51.2 (336)	31.6 (19)	21.7 (23)	28.6 (63)	70.3 (101)	50.0 (76)	59.5 (37)
The use of EFQM in implementing a quality system	29.5 (319)	20.0 (20)	60.9 (23)	3.4 (59)	57.4 (101)	4.3 (69)	20.0 (30)
Active quality improvement team(s)/circles†	349	23	24	65	106	76	37
1	27.5	47.8	4.2	7.7	19.8	42.1	48.6
2	17.5	17.4	41.7	20.0	16.0	14.5	13.5
3	38.1	30.4	29.2	47.7	53.8	23.7	13.5
4	13.2	4.3	25.0	23.1	8.5	9.2	21.6
Committee or person responsible for:							
Hospital infections control	98.9 (353)	100 (25)	100 (24)	100 (78)	100 (107)	100 (80)	89.5 (38)
Blood transfusion	92.2 (348)	100 (25)	100 (24)	100 (78)	87.5 (104)	92.1 (76)	78.9 (38)
Prevention of bed sores	85.3 (346)	66.7 (21)	95.8 (24)	79.7 (64)	94.2 (104)	78.9 (76)	84.6 (39)
Policy on use of antibiotics	92.6 (352)	87.0 (23)	95.8 (24)	96.9 (65)	98.1 (106)	96.1 (76)	66.7 (39)

*The results for the UK and the Netherlands are included in the total scores but not listed separately, due to the very low response rates.

†The sum of percentages may not always equal 100%; the percentage answers “don’t know/no answer” are not listed here. 1 =  yes, in most departments (>50%); 2 =  yes, in most departments (>50%), but not systematically; 3 =  yes, in some departments (<50%); 4 =  no.

EFQM, European Foundation for Quality Management.

### Sampling and recruitment

Our survey focused on European hospitals with a minimum of 100 acute care beds, and offering care for at least two out of the three conditions selected for study (AMI, appendicitis and deliveries). Two additional criteria defined the hospital sample: ownership status (public, private not-for-profit and private for-profit), and actual or potential cross-border care delivery.^31^ The target was to include a total of 600 hospitals covering eight countries. For smaller countries (Belgium, the Netherlands, Ireland, Czech Republic) this meant that all hospitals meeting the inclusion criteria were invited to participate. In the remaining countries, hospitals were randomly sampled from a list of hospitals that met the sampling criteria. Hospital recruitment was done by the country coordinators, who used different strategies. To make participation more appealing to hospitals, a package of advantages was offered to the hospitals, including membership in the MARQuIS network, a certificate of participation, and free subscription to the project’s newsletter. Hospitals that agreed to participate in the survey received an e-mail inviting them to enter the MARQuIS website (http://www.marquis.be) and fill out the web-based questionnaire. Upon request, a paper version of the questionnaire could be used and sent to the researchers (MJMHL or IR). Data were collected from the beginning of April to the end of August 2006. Hospitals received up to three reminders. Again, country coordinators used different approaches to raise the response rates.

### Validation of the data

To validate the questionnaire data, two analyses were performed by using data collected during on-site hospital visits in a selected sample of 89 hospitals that had previously completed the questionnaire. Visits were performed by independent external auditors. All aspects of on-site visits are described in detail elsewhere.^32^

During the visits, the hospitals’ key informants were asked to answer 25 questions that had been previously asked in the questionnaire. The reliability of the questionnaire was assessed by the level of agreement between the responses to these 25 questions as given in the questionnaire and during the on-site visits. In addition, for 14 of these 25 questions the external auditors requested evidence to check the answers given during the audit. Criterion validity was then assessed as the degree of agreement between the information provided by key informants and the evidence found to underpin this information. Reliability and validity were assessed as the index of expected agreement, which is the proportion of cases in which the results of both assessments matched.^33^

## RESULTS

### Response rates and study population

The country coordinators approached a total of 1396 hospitals, of which 483 visited the web-based questionnaire and ultimately 389 submitted the completed questionnaire (table 1). Response rates varied per country. The study population consisted of public (80%) as well as private (20%) hospitals, and included university (23.5%), teaching (48.9%) and non-teaching hospitals (27.6%). The mean number of sites per hospital was 2.46. Almost a quarter of all hospitals were considering collaboration to deliver cross-border patient care, but few hospitals were doing so at the time of the study.^15^

**Table 1:** Hospital recruitment and response rates by country

	Hospitals approached	Hospitals entering web-based questionnaire	Hospitals concluding web-based questionnaire
UK	250	41	14
Ireland	44	29	25
The Netherlands	97	12	10
Belgium	45	33	25
France	322	100	78
Spain	307	131	113
Poland	250	84	80
Czech Republic	81	53	44
Total	1396	483	389

### Results of the validation process

Regarding reliability, comparison of the information obtained for 25 items from questionnaires and on-site visits resulted in the following ranges of agreement: for five items agreement was over 90%, for 12 items the level of agreement was >70% and <90%, and for eight items agreement was <70%. Given the period of 7–9 months between the questionnaire and the on-site visits, it is likely that at least some of the items studied had actually changed. In addition, the 14 items analysed to assess criterion validity resulted in the following levels of agreement: seven items had an agreement index of >90%, six items rated >70% and <90%, and one item scored an index of <70%. Based on these results, we considered the information collected with the questionnaire a fair approximation of the actual situation at participating hospitals.

### QI strategies

A previous MARQuIS study^34^ identified seven mandatory QI strategies for hospitals. The extent to which these strategies are applied at European hospitals is reported below.

#### QI strategy 1: Organisational quality management programmes

Hospitals reported using quality management programmes in developing and implementing QI (table 2). Overall, the International Organization for Standardization (ISO) 9000 management system standards were used most often, and the European Foundation for Quality Management (EFQM) model was used least often. However, there were wide variations between countries. Belgium was the only country where the EFQM model was preferred by most hospitals (60.9%); in all other countries the ISO system was the dominant scheme. In Poland (50.0%), the Czech Republic (59.5%), and Spain (70.3%) the use of ISO was widespread. In Spain some hospitals seemed to use both schemes. Irish hospitals reported moderate use of a quality management programme (ISO = 31.6%, EFQM 20.0%), but stressed the systematic use of specific QI teams in most of their hospital departments, either systematically (47.8%) or unsystematically (17.4%). French hospitals relied least on the programmatic approach with the ISO (28.6%) or EFQM system (3.4%), and the availability if QI teams was limited (27.7%).

Responsibilities for, and policies on, blood transfusion, the use of antibiotics, and hospital infection control were clearly assigned to a committee or person in almost all hospitals across Europe, with the exception of Czech hospitals, which reported significantly less clear organisation for all three hospital-wide functions. Spanish hospitals showed a gap in organising responsibilities for blood transfusion (87.5%), and Irish hospitals in the use of antibiotics (87%). Responsibility for the prevention of bed sores seemed less structured within European hospitals, varying from a reported 66.7% in Ireland to 95.8% in Belgium.

#### QI strategy 2: Systems for obtaining patients’ views

Monitoring the views of patients by systematically conducting patient surveys was a common practice in 64.5% of the participating European hospitals. The Czech Republic stood out, reporting that 91.9% of their hospitals systematically monitored patient views. These numbers refer to hospital-wide systems for collecting patients’ views on the care they received. At the department level, patients are asked at discharge for their opinion on the quality of care delivered by the hospital staff. In France this strategy was widely implemented, with approximately 65% of the hospitals reporting a policy to measure patients’ opinion at discharge. In Poland, this was a common practice in less than 14% of the hospitals. The rates varied significantly for the three medical conditions included in our study, and variation between countries seemed greater than within countries (table 3).

**Table 3:** Quality improvement (QI) strategies as applied in European hospitals: systems for getting the views of patients (total and per country,* numbers are valid percentages†)

Specification of QI strategy	Total	Ireland	Belgium	France	Spain	Poland	Czech Republic
Monitoring the views of patients/performing patient surveys (n = 344)	344	22	23	66	104	75	37
1	64.5	54.5	60.9	56.1	67.3	61.3	91.9
2	14	18.2	13.0	13.6	11.5	14.7	8.1
3	15.7	27.3	13.0	21.2	16.3	16.0	0
4	4.7	0	8.7	7.6	4.8	5.3	0
Analysis of patient complaints (n = 350)	350	23	24	66	106	73	38
1	85.7	82.6	83.3	83.3	88.7	84.0	92.1
2	6.6	4.3	8.3	6.1	2.8	8.0	7.9
3	5.4	8.7	4.2	9.1	5.7	5.3	0
4	1.7	4.3	4.2	1.5	2.8	0	0
Patient involvement in‡:							
Discussing the results of patient surveys and complaints handling (n = 345)	345	23	24	63	105	75	38
1	7.2	4.3	0	20.6	1.9	4.0	7.9
2	6.7	4.3	8.3	19.0	1.0	4.0	2.6
3	19.1	30.4	12.5	30.2	17.1	9.3	15.8
4	60.6	52.2	70.8	22.2	74.3	76.0	68.4
The development of quality criteria/standards (n = 348)	348	23	24	65	106	75	38
1	1.7	0	0	1.5	0	1.3	7.9
2	2.3	8.7	0	2.3	0	4.0	0
3	16.1	34.8	16.7	16.9	7.5	9.3	10.5
4	73.6	52.2	70.8	72.3	86.8	77.3	76.3
Designing protocols (n = 345)	345	22	24	65	104	75	38
1	1.2	0	0	0	0	2.7	5.3
2	1.7	4.5	0	3.1	0	2.7	0
3	15.1	27.3	8.3	24.6	8.7	6.7	0
4	76.5	63.6	83.3	70.8	86.5	77.3	89.5
The evaluation of achieving quality objectives (n = 347)	347	23	24	66	104	75	38
1	4.9	0	8.3	6.1	1.0	8.0	5.3
2	7.2	13.0	8.3	9.1	1.9	10.7	2.6
3	20.5	56.5	16.7	40.9	8.7	8.0	7.9
4	61.4	26.1	58.3	42.4	82.7	61.3	78.9
Participation in (quality) committees (n = 345)	345	23	24	66	104	73	37
1	7.0	8.7	0	25.8	1.0	1.4	2.7
2	2.6	8.7	0	6.1	0	0	2.7
3	18.6	30.4	12.5	47.0	12.5	2.7	0
4	65.5	43.5	79.2	21.2	80.8	82.2	89.2
Participation in improvement projects (n = 342)	342	22	22	66	103	74	38
1	3.5	9.1	0	6.1	1.0	1.4	7.9
2	5.8	9.1	0	10.6	1.9	6.8	0
3	27.5	40.9	22.7	53.0	21.4	13.5	5.3
4	57.0	36.4	68.2	30.3	67.0	68.9	81.6
Patients’ opinion about quality of care asked at discharge (for patients with acute myocardial infarction) (n = 319)	319	22	24	49	102	69	37
1	34.5	22.7	37.5	69.4	39.2	8.7	40.5
2	15.4	13.6	12.5	12.2	12.7	15.9	27.0
3	23.8	18.2	37.5	8.2	22.5	24.6	29.7
4	20.7	31.8	8.3	6.1	22.5	43.5	2.7
Patients’ opinion about quality of care asked at discharge (for patients with appendicitis) (n = 313)	313	22	21	51	100	71	34
1	39.6	22.7	47.6	76.5	44.0	8.5	47.1
2	14.7	18.2	4.8	13.7	10.0	19.7	17.6
3	19.8	18.2	23.8	3.9	24.0	21.1	20.6
4	19.2	22.7	14.3	2.0	21.0	36.6	11.8
Patients’ opinion about quality of care asked at discharge (patients at maternal service) (n = 301)	301	18	24	50	87	73	34
1	44.9	27.8	41.7	50.0	44.8	24.7	50.0
2	14.6	11.1	16.7	11.8	14.9	20.5	11.8
3	14.0	11.1	16.7	23.5	14.9	15.1	23.5
4	15.3	22.2	20.8	2.9	17.2	24.7	2.9

*The results for the UK and the Netherlands are included in the total but not listed separately, due to the very low response rates. †The sum of percentages may not always equal 100%; the percentage answers “don’t know/no answer” are not listed here. 1 =  yes, in most departments (>50%); 2 =  yes, in most departments (>50%), but not systematically; 3 =  yes, in some departments (<50%); 4 =  no. ‡From this point on the answer categories 1–4 should be read as: 1 =  yes always; 2 =  most of the time; 3 =  sometimes; 4 =  no.

Across participating European hospitals, patient involvement seemed to be little developed. Hospitals were asked to identify the activities in which individual patients or patient organisations were always or almost always involved. Participation in the design of protocols or the development of standards was reported by 3% to 4% of all hospitals; participation in improvement projects or in quality committees was reported by less than 10%. Patient involvement was best implemented in France, with almost 40% of the hospitals reporting that they involved patients in the discussion of the results of patient surveys or complaints, and 32% stating that patients participated in quality committees.

#### QI strategy 3: Patient safety systems

Hospitals were asked how patient safety was organised and managed, whether the results were reported, and if so, how they were reported (table 4). Responsibility for patient safety was assigned to a committee or person in approximately 75% of the hospitals. At 39.1% of the hospitals a risk management programme or system was in place; 50% of the hospitals systematically reported and analysed adverse events, and 55.6% also reported complications to the medical staff. These are average numbers for Europe; variation between countries was substantial. Irish hospitals scored consistently high (>90%), and Belgium and Spanish hospitals relatively low on the availability of safety systems.

**Table 4:** Quality improvement (QI) strategies as applied in European hospitals: specific patient safety systems; total and per country*, numbers are valid percentages (total item response in absolute numbers)

Specification of QI strategy	Total		Ireland	Belgium	France	Spain	Poland	Czech Republic	
Adverse event reporting and analysis† (n = 348)	348		22	24	66	106	75	37	
1	50.0		90.9	20.8	56.1	30.2	50.7	83.8	
2	14.4		4.5	8.3	18.2	13.2	14.7	10.8	
3	28.2		4.5	58.3	22.7	43.4	25.3	5.4	
4	6.0		0	8.3	3.0	12.3	5.3	0	
Risk management programme/system† (n = 348)	348		23	24	66	106	73	38	
1	39.1		91.3	16.7	40.9	34.9	21.9	57.9	
2	13.8		4.3	4.2	16.7	9.4	15.1	23.7	
3	25.3		4.3	54.2	39.4	30.2	15.1	5.3	
4	18.7		0	16.7	3.0	23.6	38.4	13.2	
Patient safety person/group	y = 73.5 (347)		y = 95.7 (23)	y = 58.3 (24)	y = 83.1 (65)	y = 75.0 (104)	y = 54.1 (74)	y = 74.4 (39)	
Patient identification systems: use of bracelets in the emergency department	y = 39.8 (334)		y = 56.5 (23)	y = 63.6 (22)	y = 37.3 (61)	y = 38.8 (103)	y = 36.4 (77)	y = 14.7 (34)	
Patient identification systems: use of bracelets for admitted patients	y = 46.5 (333)		y = 100 (23)	y = 90.9 (22)	y = 29.5 (61)	y = 42.2 (102)	y = 35.1 (77)	y = 29.4 (34)	
Drug safety:									
Standardised limited number of drugs	y = 91.4 (348)		y = 73.9 (23)	y = 95.8 (24)	y = 84.6 (65)	y = 99.0 (105)	y = 98.7 (76)	y = 73.7 (38)	
Electronic drug prescription system	y = 39.4 (350)		y = 13.0 (23)	y = 25.0 (24)	y = 33.3 (66)	y = 38.7 (106)	y = 38.2 (76)	y = 86.8 (38)	
Expiration date checked (AMI)	y = 91.4 (315)		y = 81.8 (22)	y = 91.7 (24)	y = 84.8 (46)	y = 90.1 (101)	y = 97.1 (70)	y = 94.7 (38)	
Drugs locked (AMI)	y = 73.5 (317)		y = 81.8 (22)	y = 75.0 (24)	y = 69.6 (46)	y = 43.1 (102)	y = 98.6 (70)	y = 97.4 (38)	
HR drugs separately stored (AMI)	y = 89.9 (316)		y = 85.7 (21)	y = 91.7 (24)	y = 80.4 (46)	y = 90.2 (102)	y = 97.1 (70)	y = 84.2 (38)	
MRSA testing (AMI)	y = 6.9 (306)		y = 4.8 (21)	y = 4.2 (24)	y = 4.7 (43)	y = 0 (99)	y = 14.9 (67)	y = 5.4 (37)	
Reporting complications to medical staff		y = 55.6 (304)	y = 44.4 (18)	y = 35.0 (20)	y = 22.8 (57)	y = 63.6 (88)	y = 58.3 (72)	y = 85.7 (35)	

*The results for the UK and the Netherlands are included in the total but not listed separately, due to the very low response rates.

†The sum of percentages may not always equal 100%; the percentage answers ‘don’t know/no answer’ are not listed here. 1 =  yes, in most departments (>50%); 2 =  yes, in most departments (>50%), but not systematically; 3 =  yes, in some departments (<50%); 4 =  no. y, yes.

AMI, acute myocardial infarction; MRSA, methicillin resistant *Staphylococcus aureus*.

Specific safety questions addressed drug safety management and patient identification. In general, drug safety seemed to be assured in all participating hospitals: the use of drugs was standardised and controlled, and systems for storing, checking and preventing unauthorised access to drugs seemed well implemented. Electronic drug prescriptions were used widely only in Czech hospitals (86.8%). By comparison, in Ireland a mere 13% of the hospitals reported use of electronic prescriptions. For patient identification systems the findings were the opposite, with 100% of Irish versus 29.4% of Czech hospitals using bracelets to identify admitted patients.

#### QI strategy 4: Clinical guidelines

Clinical guidelines were widely used at participating European hospitals. Hospital-wide guidelines for preoperative assessment and prophylactic antibiotic use were in place in the vast majority (75–90%) of hospitals. In the Czech Republic and Ireland, guidelines for prophylactic antibiotic use were least widely used, versus 100% coverage in Belgian hospitals.

Laboratory work seemed to be highly standardised across the various types of laboratories across Europe. On average, standard operating procedures (SOPs) were available in approximately 90% of all hospitals. At the department level, availability of clinical guidelines or protocols was significantly less common. In summary, guidelines were available for the management of patients with AMI (mean  = 86.7%), appendicitis (mean  = 54.3%), or obstetrical problems such as breech presentation (71.5%) and vaginal birth after caesarean delivery (64.5%). Between-country variation for the availability of clinical or practice guidelines was limited for hospital-wide guidelines and SOPs, but substantial for condition-related, department-level guidelines (table 5).

**Table 5:** Quality improvement (QI) strategies as applied in European hospitals: clinical and practice guidelines; total and per country*, numbers are positive responses in valid percentages (total item response in absolute numbers)

Specification of QI strategy	Total	Ireland	Belgium	France	Spain	Poland	Czech Republic
Hospital-wide guidelines:							
Preoperative assessment	74.8 (306)	72.7 (22)	90.5 (21)	57.4 (47)	71.4 (98)	75.7 (70)	88.2 (34)
Use of antibiotics	83.0 (311)	63.6 (22)	100 (22)	88.2 (51)	82.5 (97)	92.9 (70)	51.4 (35)
Prophylactic use of antibiotics	89.6 (309)	66.7 (21)	100 (22)	94.1 (51)	94.8 (97)	91.4 (70)	71.4 (35)
Standard operating procedures for various types of laboratories:							
Clinical chemistry	93.5 (310)	95.5 (22)	100 (22)	88.5 (52)	94.7 (95)	93.8 (65)	89.5 (38)
Pathology	72.5 (265)	95.7 (23)	76.2 (21)	71.0 (31)	82.6 (92)	46.0 (50)	58.8 (34)
Microbiology laboratory	87.6 (298)	95.2 (21)	100 (22)	89.8 (49)	90.5 (95)	85.9 (64)	66.7 (33)
Pharmacy	86.9 (314)	91.3 (23)	86.4 (22)	90.2 (61)	92.7 (96)	87.7 (65)	59.4 (32)
Diagnostic radiology	89.0 (310)	86.4 (22)	100 (21)	83.9 (56)	92.6 (95)	89.7 (68)	79.4 (34)
Clinical guidelines for AMI: management of AMI patients	86.7 (316)	72.7 (22)	95.8 (24)	89.1 (46)	89.3 (103)	79.4 (68)	86.8 (38)
Clinical guidelines for appendicitis:							
Management of suspected appendicitis	54.3 (311)	36.4 (22)	47.8 (23)	52.0 (50)	49.5 (97)	67.6 (71)	51.4 (35)
Wrong site, wrong surgery	42.0 (307)	68.2 (22)	54.5 (22)	47.9 (48)	15.6 (96)	57.7 (71)	29.4 (34)
Clinical guidelines for obstetrics:							
Breech presentation	71.5 (281)	58.8 (17)	81.8 (22)	61.7 (47)	60.8 (79)	90.0 (70)	59.4 (32)
VBAC	64.5 (293)	35.3 (17)	52.0 (25)	64.6 (48)	59.0 (83)	83.3 (72)	50.0 (34)

*The results for the UK and the Netherlands are included in the total but not listed separately, due to the very low response rates.

AMI, acute myocardial infarction; VBAC, vaginal birth after caesarean section.

#### QI strategy 5: Performance indicators or measures

For the three medical conditions included in this study, hospitals were asked to report the availability of performance data for a selection of clinical indicators. Table 6 shows the findings. In summary, the availability of AMI performance data was most complete, averaging approximately 70% for the seven selected indicators. Poland reported the highest percentages, France the lowest. Performance data on the management of appendicitis were being collected for approximately 50% of the five indicators, varying from 42% for perforated appendicitis treated surgically 24 h after admission to 68.2% for wound infections. The Czech Republic and Poland performed best in this area. Lastly, the statistics for obstetrical indicators varied from 54.0% for the rate of vaginal birth after caesarean delivery to 85.3% for the percentage of caesarian deliveries. Obstetrical data were most complete in Poland and Belgium, and least complete in Ireland (table 6).

**Table 6:** Quality improvement (QI) strategies as applied in European hospitals: performance indicators or measures; total and per country*, numbers are positive responses in valid percentages (total item response in absolute numbers)

Specification of QI strategy	Total	Ireland	Belgium	France	Spain	Poland	Czech Republic
Availability of AMI performance indicators:							
Door-to-needle time	57.7 (291)	68.2 (22)	62.5 (24)	45.2 (42)	62.0 (92)	51.6 (64)	55.9 (34)
Receipt of reperfusion	70.3 (293)	54.5 (22)	75.0 (24)	61.4 (44)	66.7 (93)	79.4 (63)	76.5 (34)
Aspirin use <24 h	71.8 (291)	68.2 (22)	79.2 (24)	59.1 (44)	69.2 (91)	82.5 (63)	70.6 (34)
Prescription ACE inhibitors at discharge	67.5 (292)	63.6 (22)	73.9 (23)	53.5 (43)	59.1 (93)	84.4 (64)	67.6 (34)
Prescription of β-blockers at discharge	71.2 (288)	63.6 (22)	73.9 (23)	51.2 (43)	69.6 (92)	87.3 (63)	71.9 (32)
Prescription of aspirin at discharge	73.6 (288)	68.2 (22)	83.3 (24)	58.1 (43)	71.4 (91)	85.5 (62)	69.7 (33)
Inpatient mortality	74.4 (289)	57.1 (21)	79.2 (24)	54.8 (42)	87.0 (92)	77.4 (62)	70.6 (34)
Availability of performance indicators for the management of appendicitis:							
Prophylactic antibiotics	53.8 (290)	33.3 (21)	43.5 (23)	43.2 (44)	65.2 (92)	73.5 (68)	13.3 (30)
Negative appendectomy	46.5 (288)	33.3 (21)	43.5 (23)	42.2 (45)	45.1 (91)	53.0 (66)	50.0 (30)
Rate of lap versus open appendectomy	51.0 (286)	47.6 (21)	60.9 (23)	62.2 (45)	58.7 (92)	21.5 (65)	64.3 (28)
Perforated appendicitis operated 24 h after admittance	42.0 (283)	20.0 (20)	39.1 (23)	29.5 (44)	40.7 (91)	50.8 (63)	56.7 (30)
Wound infections	68.2 (280)	50.0 (20)	47.8 (23)	47.7 (44)	77.8 (90)	75.4 (61)	80.0 (30)
Availability of performance indicators for deliveries:							
Induced labour rate	67.5 (280)	58.8 (17)	75.0 (20)	61.7 (47)	60.5 (81)	76.8 (69)	62.5 (32)
% Caesarean sections of total deliveries	85.3 (278)	64.7 (17)	90.0 (20)	83.7 (49)	87.7 (81)	91.0 (67)	74.2 (31)
VBAC rate	54.0 (274)	35.3 (17)	50.0 (20)	44.7 (47)	55.8 (77)	67.6 (68)	51.6 (31)
Deliveries with peridural anaesthesia	71.9 (278)	52.9 (17)	85.0 (20)	83.3 (48)	81.3 (80)	58.8 (68)	54.8 (31)

*The results for the UK and the Netherlands are included in the total but not listed separately, due to the very low response rates.

AMI, acute myocardial infarction; VBAC, vaginal birth after caesarean section.

#### QI strategy 6: Internal audit, assessment of clinical standards

Medical staff performance was systematically reviewed at 50% of the participating hospitals, and peer review (site visits) was conducted at approximately 25%. Between-country variations were considerable. Belgium, Poland and the Czech Republic reported that over 60% of the hospitals performed medical staff performance reviews, versus 26.1% of Irish hospitals. However, Irish hospitals made more use of peer review (site visits) than any other European country (39.1%).

On average, 50% of the laboratories at European hospitals were periodically surveyed by an internal audit team. Percentages varied according to the type of laboratory and between countries. France reported generally low rates; in Poland internal auditing seemed broadly implemented. However, only a third of the Polish hospitals reported the results of internal audits to their governing boards, versus approximately 90% of the hospitals in the Czech Republic and Ireland. Polish hospitals more openly shared the results with their medical staffs (59.2%), but other countries reported higher percentages. Belgium was the exception here: only 40% disclosed their results to medical staff (table 7).

**Table 7:** Quality improvement (QI) strategies as applied in European hospitals: audit, internal assessment of clinical standards; total and per country*, numbers are valid percentages (total item response in absolute numbers)

Specification of QI strategy	Total	Ireland	Belgium	France	Spain	Poland	Czech Republic
Internal auditing of hospital departments† (n = 347)	347	22	24	65	105	75	38
1	35.7	22.7	16.7	16.9	21.9	53.3	76.3
2	11.0	13.6	12.5	18.5	11.4	4.0	5.3
3	40.1	63.6	54.2	56.9	52.4	16.0	13.2
4	10.1	0	12.5	7.7	14.3	14.7	2.6
Peer review/*visitatie* of hospital departments† (n = 341)	341	23	24	62	104	76	36
1	17.3	26.1	20.8	8.1	7.7	21.1	38.9
2	8.5	13.0	16.7	3.2	8.7	6.6	8.3
3	29.9	43.5	33.3	45.2	27.9	14.5	22.2
4	37.5	13.0	20.8	41.9	51.0	42.1	25.0
Medical staff performance review† (n = 345)	345	23	24	65	103	75	38
1	36.7	17.4	41.7	38.5	23.3	52.0	42.1
2	14.5	8.7	20.8	12.3	11.7	16.0	21.1
3	23.7	52.2	25.0	15.4	32.0	10.7	23.7
4	21.1	21.7	8.3	26.2	30.1	14.7	13.2
Periodical internal audits of laboratories:							
Clinical chemistry laboratory	y = 60.3 (300)	y = 68.2 (22)	y = 77.3 (22)	y = 20.0 (33)	y = 46.2 (93)	y = 78.8 (66)	y = 81.1 (37)
Pathology laboratory	y = 37.4 (254)	y = 72.7 (22)	y = 28.6 (21)	y = 3.7 (27)	y = 34.1 (88)	y = 30.6 (49)	y = 44.1 (34)
Microbiology laboratory	y = 54.3 (278)	y = 81.0 (21)	y = 81.8 (22)	y = 17.1 (41)	y = 42.4 (92)	y = 74.1 (58)	y = 51.6 (31)
Pharmacy laboratory	y = 47.5 (297)	y = 54.5 (22)	y = 18.2 (22)	y = 21.8 (55)	y = 45.1 (91)	y = 73.8 (61)	y = 50.0 (32)
Diagnostic radiology laboratory	y = 48.5 (291)	y = 60.0 (20)	y = 25.0 (20)	y = 12.2 (49)	y = 41.1 (90)	y = 72.6 (62)	y = 66.7 (36)
Results of internal audits are formally reported to:							
Hospital’s governing board	y = 65.0 (343)	y = 91.3 (23)	y = 62.5 (24)	y = 44.4 (63)	y = 79.6 (103)	y = 34.2 (73)	y = 89.7 (39)
Medical staff	y = 67.4 (304)	y = 73.7 (19)	y = 40.0 (20)	y = 72.9 (59)	y = 65.1 (83)	y = 59.2 (71)	y = 91.4 (35)

*The results for the UK and the Netherlands are included in the total but not listed separately, due to the very low response rates. †The sum of percentages may not always equal 100%; the percentage answers “don’t know/no answer” are not listed here. 1 =  yes, systematically in most departments (>50%); 2 =  yes, in most departments (>50%), but not systematically; 3 =  yes, in some departments (<50%); 4 =  no.

y, yes.

#### QI strategy 7: External assessment

Most hospitals (88%) have been assessed (at least in part) by an external organisation such as an accreditation (59.4%) or certification (49.4%) institute, a patient organisation (18.5%), or a government inspection body (66%). Some hospitals were audited by more than one organisation. In Spain, for instance, 64.8% of all hospitals (n = 88) reported being evaluated by an accreditation body, and 63.6% by a certification institute. In France (n = 63), 93.7% of all hospitals had been accredited, and in Ireland (n = 22) 90.9%. In Poland (n = 75), government inspections were the most frequently reported type of external evaluation (76%).

French hospitals reported being most open (92.3%), and Spanish hospitals the least open (19.8%) about their assessment results. On average, 52.9% of the hospitals in our sample publicly disclosed their assessment results (table 8). Most participating hospitals (84.3%) reported plans for re-evaluation within the next 3 years (not shown). Accreditation bodies were listed most frequently as the future assessors; in the Czech Republic, Ireland, the UK, and the Netherlands, more than 85% of the hospitals expressed this intention, and the figures were 78.1% for France and 77.3% for Spain.

**Table 8:** Quality improvement (QI) strategies as applied in European hospitals: external assessment, schemes and programmes; total and per country*, numbers are positive responses in valid percentages (total item response in absolute numbers)

Specification of QI strategy	Total	Ireland	Belgium	France	Spain	Poland	Czech Republic
(Part of) hospital previously externally assessed	88.0 (351)	100 (23)	95.8 (24)	96.9 (65)	85.8 (106)	82.9 (76)	71.8 (39)
External assessment by:							
Accreditation institute	59.4 (323)	90.9 (22)	20.8 (24)	93.7 (63)	64.8 (88)	35.5 (76)	29.4 (34)
Certification institute	49.4 (314)	60.0 (20)	41.7 (24)	23.3 (60)	63.6 (88)	48.6 (72)	48.6 (37)
Patient/consumer organisation	18.5 (297)	33.3 (18)	34.8 (23)	3.4 (58)	5.3 (76)	18.9 (74)	36.4 (33)
Inspection	66.0 (318)	80.0 (20)	83.3 (24)	55.9 (59)	65.9 (91)	76.0 (75)	28.1 (32)
Public disclosure of assessment results	52.9 (331)	60.9 (23)	33.3 (24)	92.3 (65)	19.8 (91)	50.6 (77)	67.6 (34)
Previous external assessment of laboratories:							
Clinical chemistry laboratory	68.6 (303)	57.1 (21)	76.2 (21)	65.2 (46)	59.4 (96)	70.8 (65)	81.6 (38)
Pathology laboratory	38.7 (261)	60.9 (23)	19.0 (21)	50.0 (28)	34.8 (92)	28.6 (49)	35.3 (34)
Microbiology laboratory	58.7 (283)	65.0 (20)	71.4 (21)	60.5 (43)	48.4 (93)	68.3 (60)	50.0 (32)
Pharmacy laboratory	54.6 (302)	72.7 (22)	59.1 (22)	65.5 (58)	39.6 (91)	62.9 (62)	39.4 (33)
Diagnostic radiology laboratory	56.8 (294)	85.0 (20)	60.0 (20)	61.2 (49)	47.3 (93)	56.5 (62)	60.0 (35)

*The results for the UK and the Netherlands are included in the total but not listed separately, due to the very low response rates.

## DISCUSSION

**Key messages**

- Quality improvement (QI) strategies are widely used in European hospitals. The most widely applied QI strategy is external assessment of hospitals, whereas patient involvement in QI activities is the least widely applied
- Reported implementation varies per country. This leaves considerable room for progress in making QI in hospitals a reality
- Differences also suggest that, for various reasons, countries may prefer some QI strategies over others

**Contribution to better patient care**

- International comparisons of the use of QI strategies can promote learning and the spread of good practice
- The results of this study may be useful to national policy makers in monitoring the attainment of healthcare policy goals

**Points for further research**

Further research should focus on exploring the relationship between the use of QI strategies and the actual performance of hospitals, including the relative contribution of each of the seven QI strategies to performance

This study has some limitations. The response rates varied per country, and were particularly low in the UK and the Netherlands. This may be explained by the various approaches used to recruit hospitals for the MARQuIS project, and to the effect of questionnaire fatigue due to the over-application of questionnaire surveys to evaluate healthcare performance in general. We therefore cannot rule out participation bias. Also, accuracy of the information is always a limitation when using self-reported data. However, the results of our validation process strongly suggest that the reported results are fairly accurate. Further, translation of the questionnaire, the use of jargon, and the involvement of people from various healthcare systems may have caused differences in how the items were interpreted. Lastly, hospitals may use local QI approaches or tools not included in this questionnaire, in which case the application of QI strategies, as described in this article, may misrepresent the “maturity” of hospitals’ quality management systems. These limitations should be taken into account when interpreting results.

International comparisons can promote learning and the spread of good practice, and are one of the ways in which the European Community is expected to raise healthcare quality. This study of how European hospitals apply seven common QI strategies found considerable variation between the level of implementation of the different strategies—a finding that leaves considerable scope for progress in making QI a reality.

The use of QI strategies at the European level was determined or at least influenced by national and international policy making and regulation, as well as by national and local bottom-up actions initiated by professionals or others.^35^ In our study 88% of all hospitals reported having been externally assessed; the widespread application of the “external assessment” QI strategy can be ascribed to the fact that most countries have adopted one or more models of external assessment (ie, accreditation, certification or licensure) to ensure and improve hospital performance, which in turn has been related to financing healthcare delivery.

However, policies and regulations may not always be effective, as shown by the fact that in most hospitals (>90%), patient involvement in QI activities was lacking. This was despite the various legal and other efforts undertaken by the European Commission over the past decades to increase citizens’ participation in QI, and in the organisation and structure of health services in general.^4^ ^5^ Future research should focus on detecting barriers to the implementation of these QI strategies. In this regard, efforts by the EU to facilitate improvements and foster European collaboration may help to further increase implementation.^36^ ^37^

Legislation recently proposed by the European Commission stresses the values and principles of safe, high-quality health services that underpin European health systems. However, the question arises as to how these agreed-upon values and principles can be applied by member states.^36^ We believe our results may help national policy makers to monitor the attainment of healthcare policy goals. The application of more QI strategies, however, may not necessarily imply more positive effects on performance. Our findings would be even more valuable if the demonstrated use of QI strategies could be related to actual performance in hospitals. This would give EU policy makers direct input for monitoring the development of healthcare policies and regulations. Elsewhere in this supplement this relationship is explored in greater depth.
